# CSF-contacting neurons regulate locomotion by relaying mechanical stimuli to spinal circuits

**DOI:** 10.1038/ncomms10866

**Published:** 2016-03-07

**Authors:** Urs Lucas Böhm, Andrew Prendergast, Lydia Djenoune, Sophie Nunes Figueiredo, Johanna Gomez, Caleb Stokes, Sonya Kaiser, Maximilliano Suster, Koichi Kawakami, Marine Charpentier, Jean-Paul Concordet, Jean-Paul Rio, Filippo Del Bene, Claire Wyart

**Affiliations:** 1 Institut du Cerveau et de la Moelle épinière, Paris 75013, France; 2 UPMC Univ. Paris 06, Paris 75005, France; 3 Inserm UMR 1127, Paris 75013, France; 4 CNRS UMR 7225, Paris 75013, France; 5 Muséum National d'Histoire Naturelle, Paris 75005, France; 6 Neural Circuits and Behaviour Group, University of Bergen, Bergen 5008, Norway; 7 Division of Molecular and Developmental Biology, National Institute of Genetics and Department of Genetics, Sokendai (The Graduate University for Advanced Studies), Mishima, Shizuoka 411-8540, Japan; 8 Institut Curie, Paris 75005, France; 9 CNRS UMR 3215, Paris 75005, France; 10 Inserm U 934, Paris 75005, France

## Abstract

Throughout vertebrates, cerebrospinal fluid-contacting neurons (CSF-cNs) are ciliated cells surrounding the central canal in the ventral spinal cord. Their contribution to modulate locomotion remains undetermined. Recently, we have shown CSF-cNs modulate locomotion by directly projecting onto the locomotor central pattern generators (CPGs), but the sensory modality these cells convey to spinal circuits and their relevance to innate locomotion remain elusive. Here, we demonstrate *in vivo* that CSF-cNs form an intraspinal mechanosensory organ that detects spinal bending. By performing calcium imaging in moving animals, we show that CSF-cNs respond to both passive and active bending of the spinal cord. In mutants for the channel Pkd2l1, CSF-cNs lose their response to bending and animals show a selective reduction of tail beat frequency, confirming the central role of this feedback loop for optimizing locomotion. Altogether, our study reveals that CSF-cNs constitute a mechanosensory organ operating during locomotion to modulate spinal CPGs.

## Abstract (web summary)

CSF-contacting neurons are known to project to locomotor CPGs although their relevance to active locomotion is unclear. Here, the authors show that these cells constitute a mechanosensory organ relying on PKD2L1 channels to detect spinal cord curvature and modulate locomotor frequency in freely moving animals.

## Graphical abstract

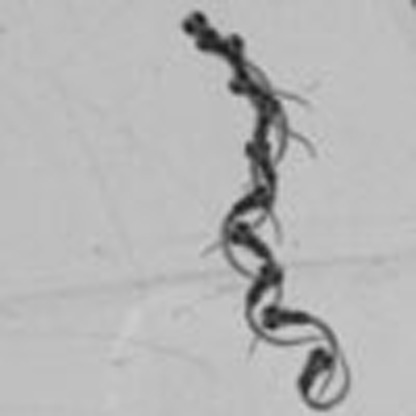

Behavior has long been known to be modulated by the content of cerebrospinal fluid (CSF)^1^,^2^. Studies of CSF flow have shown that locomotor defects observed in normal pressure hydrocephalus can be reversed by CSF elimination. More recently, CSF has been shown to relay important clues to migrating neurons during development^3^,^4^ and to remove metabolites during sleep^5^. However, the mechanism by which molecular and hydrodynamic properties of the CSF change network dynamics is unknown. In the vertebrate spinal cord, a unique and conserved population of GABAergic neurons extends microvilli into the CSF^6^,^7^,^8^,^9^,^10^. These neurons, referred to as CSF-cNs (and in *Xenopus* and *Danio rerio* as KA neurons^11^), are among the most poorly understood components of the vertebrate motor network. We previously showed that CSF-cNs can modulate locomotion^12^. Recently, we demonstrated that these neurons perform this modulation by projecting onto essential glutamatergic components of the locomotor central pattern generators^13^ (CPGs). *In vitro* studies show that in the spinal cord, CSF-cNs selectively express the transient receptor potential channel PKD2L1, a molecular component of the response to pH and osmolarity^14^,^15^,^16^. However, it is unknown whether these neurons *in vivo* relay sensory information onto motor circuits during innate locomotion. Here, we combine calcium imaging in moving zebrafish larvae with quantitative behavioural analysis to investigate CSF-cN activity during locomotion and impact on behavior. We show that CSF-cNs respond to both passive and active bending of the spinal cord *in vivo*. As the PKD2L1 channel is specific to CSF-cNs in the spinal cord, we investigated the role of this channel in the sensory functions of CSF-cNs *in vivo*. In *pkd2l1* mutants, cellular morphology is unaltered but the response to bending is abolished, suggesting that this channel is necessary for CSF-cN mechanosensory response to bending. Furthermore, the loss of CSF-cN sensory function leads to a reduction of tail beat frequency in *pkd2l1* mutants as well as in animals with impaired synaptic release from CSF-cNs, confirming the importance of this sensory feedback loop in the regulation of locomotion. Together, our results demonstrate that Pkd2l1 orchestrates a feedback loop that relays information on local curvature of the spinal cord to motor circuits in order to regulate the frequency of locomotion.

## Results

### CSF-cNs exhibit a motile kinocilium and multiple microvilli

**Figure 1:**
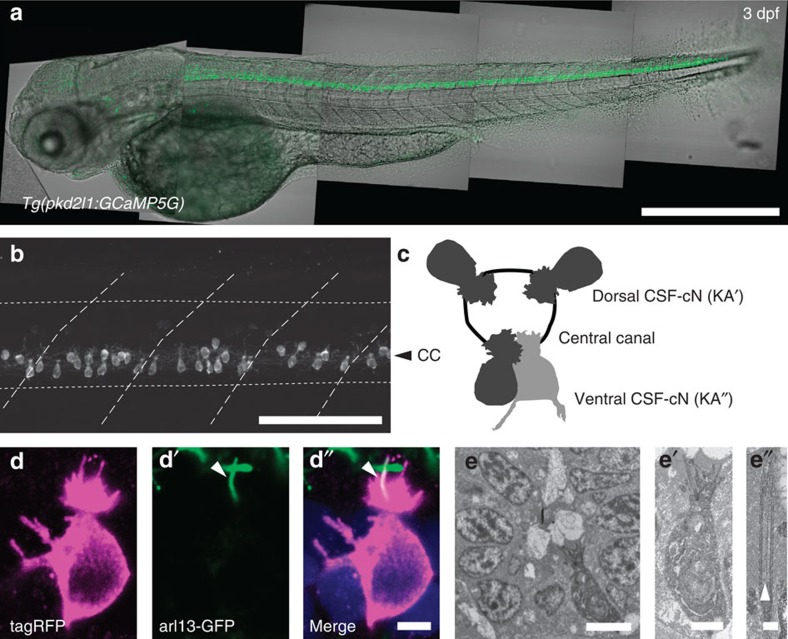
In the spinal cord, *pkd2l1*+CSF-cNs project an apical extension consisting of one kinocilium and a brush of microvilli into the central canal. (**a**) Transmitted and fluorescent image showing that the *pkd2l1* promoter in *Tg(pkd2l1:GCaMP5G)* transgenic larva drives GCaMP5G expression in CSF-cNs along the entire spinal cord. Scale bar: 500 μm. (**b**) A close up from a lateral view in the same transgenic animal shows the morphology of CSF-cNs as elongated cells with an apical extension reaching the central canal. Scale bar: 100 μm. (**c**) Schematic depicting dorsal and ventral CSF-cN location around the central canal. (**d**) Confocal microscopy of 50 μm sections of the 4 dpf triple transgenic larva *Tg(pkd2l1:gal4, UAS:tagRFP-CAAX;cmcl2:GFP, βact:Arl13-GFP)* shows that CSF-cNs project one Arl13-GFP+ cilium (arrowhead) and multiple microvilli into the CSF. Scale bar: 3 μm. (**e**) Transmission electron microscopy in *Tg(pkd2l1:Gal4)* larvae injected with UAS:APEX2-tagRFP shows a single ventral CSF-cN reaching the central canal (close up in (**e′**), scale bar: 2 μm) and exhibiting a motile 9+2 cilium (arrowhead, (**e″**), scale bar: 250 nm).

We used a promoter of *pkd2l1* that specifically targets CSF-cNs in the larval spinal cord (Fig. 1a, out of 253 *pkd2l1*^*+*^ cells from five larvae, CSF-cNs represented 94.2% of total cell counts, see also^13^) to drive the expression of fluorescent proteins (Fig. 1a–e). Previous studies suggested that CSF-cNs extend cilia into the central canal^6^,^9^,^11^,^17^,^18^ as depicted in Fig. 1c. We co-labeled CSF-cNs with Arl13b–GFP as a marker of the ciliary axoneme in *Tg(βact:arl13-GFP, pkd2l1:Gal4;cmcl2:eGFP, UAS:tagRFP-CAAX)* triple transgenic larvae (Fig. 1d). We observed a single cilium extending from a brush of microvilli (Fig. 1d′). To establish whether the CSF-cN cilium was motile, we used APEX-2, a genetically-encoded peroxidase, to label CSF-cNs for electron microscopy (Fig. 1e). Our analysis of the ciliary ultrastructure reveals a 9+2 organization, indicating that CSF-cNs extend a single motile cilium into the central canal (Fig. 1e′). This was confirmed by video recording of CSF-cNs *in vivo* (Supplementary Movie 1) as well as in zebrafish dissociated cell cultures (Supplementary Movie 2). The unusual morphology of CSF-cNs may be described as disorganized hair cells located at the interface with the CSF and extending an ascending ipsilateral axon in the spinal cord.

### CSF-cNs show minimal activation during fictive escapes

**Figure 2:**
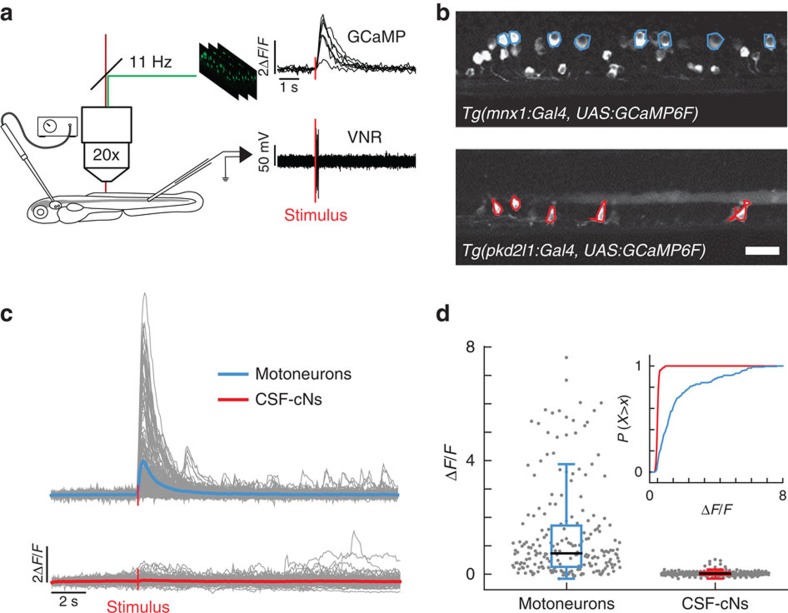
CSF-cNs are minimally activated during fictive escapes when no muscle contraction occurs. (**a**) Schematic view of the experimental setup combining 2-photon laser scanning microscope for calcium imaging and electrophysiological recordings of the ventral nerve root. 10 ms-long water jets delivered to the otic vesicle triggered fictive escapes in paralyzed larvae. (**b**) Lateral view showing expression of GCaMP6f in MNs in the double transgenic larva *Tg(mnx1:Gal4, UAS:GCaMP6f;cryaa:mCherry)* and in CSF-cNs in *Tg(pkd2l1:gal4, UAS:GCaMP6f;cryaa:mCherry)*. ROIs indicate cells included in the analysis. Only the dorsalmost MNs were analyzed in the *mnx1* line. Scale bar: 20 μm. (**c**) Typical calcium transients recorded in MNs (blue) and in CSF-cNs (red) during fictive escapes; ‘stimulus' indicates when the water jet was triggered, average response in coloured lines. (**d**) Quantification of calcium transient amplitude in MNs and CSF-cNs (each data point represents one recording from one cell; plots use median as the measure of central tendency; inset is the cumulative histogram of calcium responses). Responses in both populations are greater than baseline (204 MNs from 6 larvae: mean Δ*F*/*F*=1.2, *P*<1.0 × 10^−8^; 192 CSF-cNs from 7 larvae: mean Δ*F*/*F*=0.042, *P*=3.78 × 10^−8^), but CSF-cNs exhibit significantly less activity than MNs (*P*=0.014).

To investigate the involvement of CSF-cNs in the control of locomotion, we characterized their activity patterns during spinal network activity in paralyzed larvae. We used two-photon calcium imaging to record cellular activity during fictive escape behavior induced by a water jet to the otic vesicle (Fig. 2a). Fictive locomotor output was monitored by recording the ventral nerve root (Fig. 2a)^19^. We compared CSF-cN activity to dorsal motor neurons (MNs), which are known to be active during escape behavior (Fig. 2b)^20^. MNs showed strong calcium transients during fictive escape behavior (mean Δ*F*/*F*_MNs_=1.2±0.1; Fig. 2c top panels and 2d). In contrast, fictive locomotion only led to minute changes in calcium activity in CSF-cNs (mean Δ*F*/*F*_CSF-cNs_=0.042±0.007; Fig. 2c,d bottom panels). To verify that the lack of activity in CSF-cNs was not due to α-bungarotoxin used as a paralytic, we repeated the experiment in immotile *cacnb1*^*ts25/ts25*^ larvae, where a mutation in the β_1a_ subunit of the dihydropyridine receptor prevents contraction of skeletal muscle^21^. The lack of activation of CSF-cNs during fictive escapes in *cacnb1*^*ts25/ts25*^ mutants (Supplementary Fig. 1) confirms that CSF-cNs are minimally activated when muscles are not contracting.

### Muscle contraction leads to strong activation of CSF-cNs

**Figure 3:**
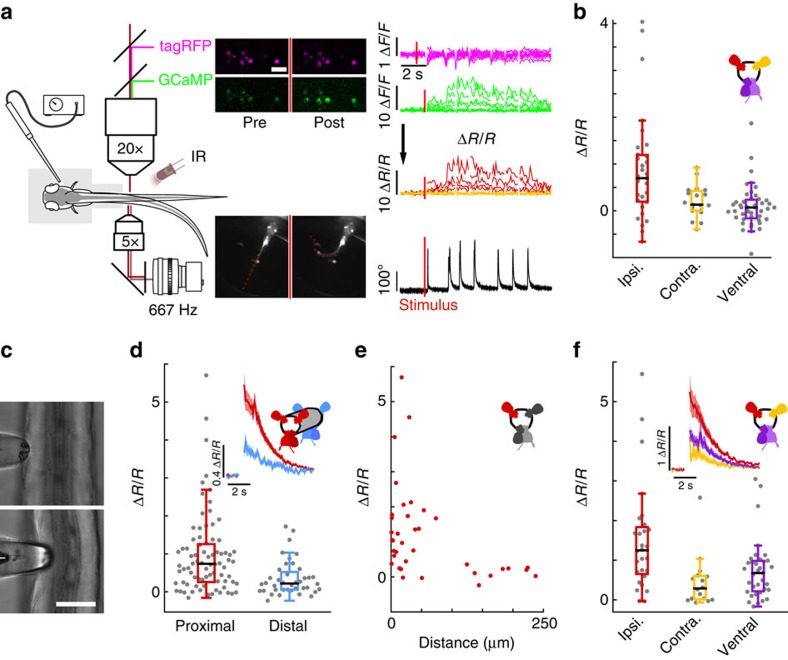
CSF-cNs respond to active muscle contraction as well as to passive mechanical bending of the spinal cord. (**a**) Schematic describing 2-photon imaging experiments used to record simultaneously from CSF-cNs expressing tagRFP (magenta) and GCaMP5 (green) in head-embedded *Tg(pkd2l1:GCaMP5, pkd2l1:tagRFP)* larvae. Infrared illumination combined with high-speed video recording shows unidirectional tail deflections induced by a water jet to the otic vesicle. Sample traces for Δ*F*/*F* of tagRFP and GCaMP shown with tail deflection during escape (note: vertical scale is 10 times larger for GCaMP compared to tagRFP signals). Subtracting the tagRFP signal from the GCaMP signal removed motion artifacts; breaks in the trace arise from frames when cells escaped from the focal plane. Scale bar: 10 μm. (**b**) Quantification of calcium transient amplitude in response to muscle contraction (*n*=11 larvae) in dorsal CSF-cNs either ipsilateral (red, 31 cells) or contralateral (yellow, 19 cells) or ventral (purple, 44 cells). Only dorsal ipsilateral cells exhibited responses greater than baseline (*P*=9.51 × 10^−4^) and all other cell types responded significantly less than dorsal ipsilateral cells (dorsal contralateral: *P*=2.43 × 10^−3^, ventral: *P*=4.85 × 10^−5^). (**c**) Passive mechanical stimulation of CSF-cNs in paralyzed larvae (*n*=5) was implemented with mechanical pressure exerted by pushing a glass probe laterally against the fish tail. Scale bar: 50 μm. (**d**) Response of proximal (<100 μm) and distal (>100 μm) CSF-cNs. Inset: average calcium response of proximal (red) versus distal (blue) CSF-cNs. (**e**) Response of dorsal ipsilateral (red) CSF-cNs as function of distance from the probe. (**f**) Response of dorsal ispsilateral (red, 28 cells), dorsal contralateral (yellow, 16 cells) and ventral (purple, 36 cells) CSF-cNs relative to the location of mechanical stimulation. Inset: Average calcium response of dorsal ispsilateral versus dorsal contralateral and ventral CSF-cNs. All cell types show a response different from 0 (dorsal ipsilateral: *P*<1.0 × 10^−8^, dorsal contralateral: *P*=5.95 × 10^−5^, ventral: *P*<1.0 × 10^−8^) and all other cell types responded significantly less than dorsal ipsilateral cells (dorsal contralateral: *P*=1.06 × 10^−3^, ventral: *P*=7.22 × 10^−3^).

To test whether CSF-cNs are recruited during active locomotion, we performed calcium imaging in the spinal cord of head-restrained, tail-free animals performing escape behaviours (Fig. 3a). We took advantage of the slow decay of GCaMP5G transients in CSF-cNs (*τ*_CSF-cNs_=1.18±0.07 s) relative to tail movements (<200 ms) to compare calcium signals before and after active tail motion. TagRFP was co-expressed in CSF-cNs with GCaMP5G to track moving cells and to correct for motion artifacts (see Methods section; Fig. 3a′ and Supplementary Fig. 2). We simultaneously performed high-speed video recording of the entire larva to quantify the behavior in response to otic vesicle stimulation (Supplementary Movie 3). In contrast to paralyzed larvae, we observed strong activation of CSF-cNs in response to tail bends (Δ*R*/*R*_CSF-cNs_=0.38±0.08). Moreover, after unilateral tail bends, our results revealed a clear activation of dorsal CSF-cNs ipsilateral to the contracting side (Δ*R*/*R*_iCSF-cNs_=0.9±0.2; Fig. 3b) whereas dorsal contralateral and ventral cells remained mostly silent (Δ*R*/*R*_cCSF-cNs_=0.20±0.08; Δ*R*/*R*_vCSF-cN_=0.10±0.07; Fig. 3b). Our data indicate that dorsal CSF-cNs respond to active bending of the spinal cord selectively on the contracting side.

### Bending of the tail recruits CSF-cNs in paralyzed animals

To test whether CSF-cNs respond to mechanical stimulation during bending of the spinal cord, we mimicked the mechanical deformation associated with muscle contraction by imposing a local bend on the tail of paralyzed larvae using a mechanical probe. The tail of agarose-embedded larvae was exposed on one side and pressed with a glass probe (Fig. 3c). Here, CSF-cNs showed activity in response to mechanical stimulation (Δ*R*/*R*_CSF-cNs_=1.0±0.1; Fig. 3d). The response was restricted to the site of stimulation and its amplitude decreased with increasing distance from the probe (Fig. 3e). Dorsal CSF-cNs ipsilateral to the stimulation site showed the largest responses (Δ*R*/*R*_iCSF-cNs_=1.5±0.3; Fig. 3f) while dorsal contralateral and ventral CSF-cNs showed a significant, albeit much smaller, response (Δ*R*/*R*_cCSF-cNs_=0.5±0.2; Δ*R*/*R*_vCSF-cNs_=0.8±0.1; Fig. 3f). Results from passive bending further support the hypothesis that CSF-cNs constitute an *in vivo* mechanosensory system locally detecting bending of the spinal cord.

### Bending-evoked responses are abolished in *pkd2l* mutants

**Figure 4:**
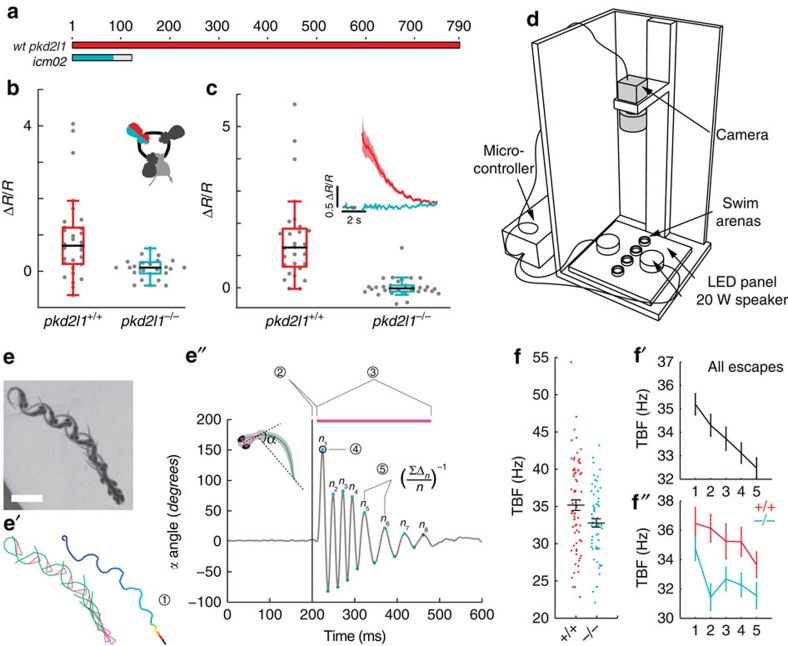
Mutation of *pkd2l1* abolishes CSF-cN response to active and passive spinal bending and leads to a reduction of swimming frequency during the escape. (**a**) The TALEN-mediated 8 nucleotide deletion in exon two of *pkd2l1* leads to a frame shift and a premature stop in *pkd2l1*^*icm02*^. (**b**,**c**) Calcium transients in dorsal ipsilateral CSF-cNs are abolished in *pkd2l1* mutant after mechanical stimulation during active (**b**) and passive (**c**) tail movement (same stimulation paradigms as used in Fig. 3, wildtype data sets for (**b**,**c**) are the same as in Fig. 3). Mutants (10 fish, 29 cells) in b show no response different from 0 (*P*=*0.13*) and are different from wildtypes (11 fish, 31 cells, *P*=1.84 × 10^−2^). Mutants (7 fish, 30 cells) in **c** show no response different from 0 (*P*=0.22) and are different from wildtypes (5 fish, 28 cells, *P*=2.79 × 10^−4^). (**d**) Experimental setup monitoring at high speed the escape response of freely swimming zebrafish larvae isolated in separated swim arenas triggered by 10 ms, 500 Hz acoustic stimuli. Acoustic stimuli were repeated five times per larva with 2 min inter trial interval. (**e**) Superimposed images showing a typical acoustic escape, (**e′**) tracking corresponding to the same escape, swim distance (1) is derived from swim bladder position. (**e″**) kinematic analysis relied on the measure of the tail angle over time *α*(*t*) enabling to measure latency (2), escape duration (3), C-bend amplitude (4) and number of oscillations and TBF based on detection of subsequent peaks (5). Speed is derived from swim distance divided by duration. Scale bar: 2 mm. (**f**) Reduction of TBF in the *pkd2l1* mutant. Each point corresponds to a value per larva averaged over multiple trials (*P*=0.0091). (**f′**) TBF decreased across trials in our paradigm (*P*=1.7 × 10^−5^). (**f″**) The reduction of TBF in the *pkd2l1* mutant (blue) compared to wild type is noticeable across trials (*P*=0.039).

The restricted expression of PKD2L1 to CSF-cNs in the spinal cord and its conservation across multiple vertebrate species suggest an important role specific to CSF-cNs^7^,^15^. To test whether this channel contributes to the mechanical response of CSF-cNs, we used transcription activator-like effector nucleases (TALENs) to generate a *pkd2l1* null mutant (Fig. 4a). CSF-cNs without functional Pkd2l1 show no overall morphological defects in zebrafish larva and their cilia were still beating *in vitro* (*n*=3). However, the responses of dorsal ipsilateral CSF-cNs to active bending (*pkd2l1*^*icm02/icm02*^ Δ*R*/*R*_CSF-cN_=0.07±0.06; Fig. 4b) and passive bending (*pkd2l1*^*icm02/icm02*^ Δ*R*/*R*_CSF-cN_=0.03±0.06; Fig. 4c) abolished in *pkd2l1*^*icm02/icm02*^ mutants. Our data indicate a crucial role of Pkd2l1 in mediating CSF-cN detection of spinal bending in zebrafish larvae.

### *pkd2l1* mutants show reduced tailbeat frequency

The ultimate output of sensory processing in the central nervous system is behavior. To probe the active contribution of the CSF-cN sensory motor loop to locomotion, we quantified acoustically-induced escape behavior in *pkd2l1*^*icm02/icm02*^ mutants, where CSF-cN response to bending is impaired (457 escapes from 141 larvae, seven experiments, Fig. 4d). Kinematic analysis revealed a specific decrease of mean tail beat frequency (TBF) in *pkd2l1*^*icm02/icm02*^ mutants compared to WT siblings (32.8±0.6 Hz versus 35.2±0.7 Hz; Fig. 4d and Supplementary Fig. 3a). We observed a significant effect of trial number on TBF (Fig. 4f), latency, distance, speed and number of oscillations (Supplementary Fig. 4b) indicating some habituation to stimulus even at an inter trial interval of 2 min. Accounting for this trial effect and using a mixed linear model, we found that TBF was reduced in the *pkd2l1*^*icm02/icm02*^ mutant across trials (Fig. 4f″). The specific reduction of TBF in larvae lacking CSF-cN sensory response reveals that CSF-cN feedback is needed to maximize locomotor frequency during active locomotion. We recapitulated this effect by silencing the vesicular release of CSF-cNs with Botulinum toxin light chain in *Tg(pkd2l1:gal4;UAS:BoTxBLC-GFP)* transgenic larvae (36.3±0.7 Hz versus 38.9±0.5 Hz; Supplementary Fig. 4 (ref. 22)). The convergence of effects obtained from these two independent methods for silencing CSF-cNs demonstrates their central role in the modulation of locomotor frequency.

## Discussion

The hypothesis that CSF-cNs constitute a mechanosensitive organ was already formulated by Kolmer, although to-date no experiments had formally addressed this question *in vivo*. Here, we report activation of CSF-cNs in response to active as well as passive spinal bending (Supplementary Fig. 5). In natural conditions, we show here that CSF-cN mechanosensory response shapes innate locomotion. Although a small population of *pkd2l1* expressing neurons was described in dorsal spinal neurons at embryonic stages^7^, this population is undetectable in the larva and therefore unlikely to mediate the behavioural effects presented here (see Supplementary Fig. 1 of reference^13^).

Whether CSF-cNs are the direct mechanoreceptors or whether they receive input from other, unidentified mechanosensitive cells remains to be determined. CSF-cNs may also respond to chemical cues released in the central canal as a response to mechanical stimulation or muscle contraction. Although we cannot exclude a chemical hypothesis, it is unclear how a chemical signal would translate into the lateralized mechanoresponse described here. Ciliary calcium signalling may contribute to the sensory response of CSF-cNs as the PKD2L1 channel contributes to ciliary signalling^23^. PKD2L1 positive CSF-cNs were previously shown to be pH sensitive^14^,^15^,^24^, even though acid sensing ion channels (ASICs) carry most of the proton current^14^,^25^,^26^. Our study here focused on the role of Pkd2l1 as, in the spinal cord, the channel is specific to CSF-cNs. However, as recently shown, PKD2L1 can trigger large calcium spikes upon changes of extracellular calcium, pH or membrane potential^24^. This channel in CSF-cNs may therefore act in concert with other channels such as ASICs contributing to mouse and lamprey CSF-cN response *in vitro*^14^,^16^,^27^ in order to generate the bending response reported here *in vivo*.

Previously, we demonstrated that spinal CSF-cNs project directly onto locomotor CPGs involved in slow locomotion^12^,^13^. During active locomotion, when muscles contract, we show that CSF-cNs can provide a proprioceptive GABAergic feedback that finely tunes the oscillatory frequency of the locomotor CPG. Further studies linking CSF-cNs to components of the fast locomotor CPG, and the escape circuit in particular, will be necessary to establish the neuronal basis for the modulation we report here. Although sensory feedback is not necessary for the oscillations, there are multiple lines of evidence suggesting that mechanosensory feedback can entrain the rhythm^28^,^29^ and gate locomotor transitions^30^. The increased frequency of oscillations due to mechanosensory feedback observed here could contribute to the massive reduction of locomotor frequency in fictive compared to active locomotion observed in earlier studies^31^,^32^.

Given that the CSF is a rich source of neuromodulators and endocrine signals, it could regulate the spontaneous firing of CSF-cNs via changes in pH or osmolarity via PKD2L1^14^,^15^,^16^,^26^ or via ATP signalling at the P2X2 receptor^17^. Tuning the spontaneous activity of CSF-cNs could modulate spontaneous locomotion in a similar manner as has recently been shown for sensory modulation of arousal locomotion in *C. elegans*^33^. Altogether, our data demonstrate a novel role for CSF-cNs in the vertebrate spinal cord and opens new perspectives on a CSF sensory interface regulating locomotion.

## Methods

### Animal care

Animal handling and procedures were validated by the Institut du Cerveau et de la Moelle épinière, Paris and the French National Ethics Committee (Comité National de Réflexion Ethique sur l'Expérimentation Animale-Ce5/2011/056) in agreement with European Union legislation. Adults were reared at a maximal density of 8 animals per liter in a 14/10 (light/dark) cycle environment. Fish were fed live artemia twice a day and feeding regime was supplemented with solid extracts matching the fish developmental stage (ZM Systems, UK). Larvae were raised at 28.5 °C with a 14/10 day/night light cycle. All experiments were performed at room temperature on 5–6 dpf larvae unless stated otherwise.

### Generation of transgenic lines

Transgenic lines used in this study are listed in Extended Data Table 1. To generate the *Tg(pkd2l1:Gal4)icm10* transgenic line expressing the Gal4 reporter gene in *pkd2l1* expressing cells, we amplified 3.8 kbp of genomic sequence immediately upstream of the predicted ATG site for the zebrafish *pkd2l1* gene (ENSDARG00000022503)^13^ as well as 630 bp of highly conserved DNA in intron 2. Both DNA fragments were then subcloned into the pT2KhspGFF plasmid using restriction digestion and T4 ligation. The 3.8 kb promoter region was cloned upstream the Gal4 (GFF) sequence in place of the *hsp* promoter, while the intronic sequence was placed after the Gal4 stop codon and before the SV40 poly-A site. For establishing the *Tg(pkd2l1:GCaMP5G)icm07*, the same strategy was used by regular cloning with the PT2 vector containing the GCaMP5G construct. *Tg(UAS:GCaMP5G)icm08* was generated by regular cloning in the PT2–14xUAS vector. The transgenic line *Tg(UAS:GCaMP6F, cryaa:mCherry)icm06* was generated by subcloning GCaMP6f^34^ into pDONR221 and then assembled into the final expression vector in a three-fragment gateway reaction (Invitrogen) using p5E-14XUAS, pME-GCaMP6f, p3E-poly(A) and pDest-cryaa:mCherry. The *Tg(pkd2l1:tagRFP)icm17* was generated from pkd2l1:GCaMP5G by swapping the GCaMP cassette for TagRFP using restriction digest/ligation as above. To generate a GFP reporter plasmid that expresses zebrafish optimized Botulinum Toxin Light Chain (BoTxLC) under Gal4/UAS control, we used an expression vector containing the Gal4-target sequence, 5x UAS, followed by an atg-less GFP. A gateway-compatible vector pT2UASMCSGW-R1R2 (5,419 bp) was used to generate the plasmid pT2UAS:BoTxLC-GFP encoding an in-frame fusion of GFP at the C-terminus of BoTxLC. The generation and validation of the UAS:BoTxLC-GFP line including the electrophysiological characterization that GABA vesicular release is abolished in *Tg(pkd2l1:gal4;UAS:BoTxLC-GFP)icm21* transgenic larvae is detailed in a related publication^22^. The *Tg(mnx1:gal4)icm23* line was based on the injection of the *mnx1* construct previously described^35^. Stable transgenic lines were established as previously described^36^. Full complement of transgenic lines generated and used is shown in Supplemental Table 1.

### Generation of *pkd2l1* mutant

RNA coding for each TALEN monomer (left arm target: 5′-TGAGAAAGGATACAAAAG-3′; right arm target: 5′-TTGAGATTTGCTTTGACG-3′) was synthesized by the mMessage mMachine *in vitro* transcription kit (Invitrogen) and injected into *Tg(pkd2l1:GCaMP5G)icm07* embryos at the one-cell stage. Injected embryos were pooled and a crude DNA extract was harvested. A DNA cassette containing the target site was amplified by PCR (primers: 5′-AGGGCAAGAGAATGGCAAGACG-3′ and 5′-TGTGTGCTAGGACTGTGGGG-3′), and the resulting band was digested by SacI (Roche) to confirm disruption of the TALEN site. The mutant alleles were cloned by TOPO cloning (Invitrogen) and sequenced to determine the exact nature of the mutations. The *pkd2l1*^*icm02*^ mutant allele is an 8 bp deletion in exon 2 causing a +1 frameshift that terminates in a stop codon within 35 amino acids of the substitution, upstream of the first transmembrane domain of Pkd2l1. All analysis was performed blind to genotype, which was only assayed at the conclusion of each experiment.

### Fluorescent immunohistochemistry

Larvae were fixed in 4% PFA +3% sucrose for 2 h at RT followed by 3X 5 min washes in PBS. After removing the skin, larvae were embedded in 4% low melting agarose and cut in 50 μm sagittal sections. Sections were mounted on a slide and blocked for 1 h in blocking solution (10% NGS, 1% DMSO, 0.5% Triton X100 in 0.1 M PBS). Slides were incubated with primary antibody over night at RT or for two days at 4 °C (1% NGS, 1% DMSO, 0.5% Triton X100 in 0.1 M PBS). After washing three times for 5 min in 0.1 M PBS with 0.5% TritonX100 (PBST), slides were incubated in the dark with the secondary antibody in PBST. After washing again three times for 5 min in PBST, mounting medium was added and slices were imaged on a confocal microscope using a 63X objective (FV-1000, Olympus). Antibodies used here were Anti-GFP (Abcam ab13970, dilution 1:500), anti-TagRFP (Life Technologies R10367, dilution 1:500), Alexa Fluor 568 goat anti-rabbit IgG (Invitrogen A11011, dilution 1:1,000), Alexa Fluor 488 goat anti-chicken IgG (Invitrogen A11039, dilution 1:1,000); all immunohistochemical samples were counterstained with DAPI (Invitrogen D3571, dilution 1:2,000).

### Electron microscopy

To label CSF-cNs for electron microscopy, we injected 60 ng μl^−1^ UAS:APEX2-tagRFP^37^ into *Tg(pkd2l1:Gal4)icm10* embryos at one-cell stage. 2.5 dpf larvae were then selected for CSF-cNs expression and euthanized in 0.2% tricaine methanesulfonate (MS-222, Sigma-Aldrich, USA) prior to fixation in 2% glutaraldehyde in 100 mM sodium cacodylate. Briefly, whole embryos were shortly rinsed in Na-cacodylate, to which glycine was added, and rinsed again in Na-cacodylate buffer. The peroxidase was further revealed in DAB (0.5 mg ml^−1^), to which 10 mM hydrogen peroxide was added^37^. After 5 min, the reaction was stopped by rinsing embryos in Na-cacodylate before postfixation in 2% osmium tetroxide and processed for standard electron microscopy. For sectioning, embryos were oriented in the coronal plane. Stained cells were identified in a widefield microscope and regions of interest serially cut on copper grids to be further observed in a HITACHI 120 kV HT 7700 electron microscope.

### Calcium imaging setup

5–6 dpf larvae were anesthetized in 0.02% MS-222 diluted in fish facility water and then mounted in glass-bottom dishes (MatTek, Ashland, Massachusetts, USA) filled with 1.5% low-melting point agarose. Calcium imaging was performed on a two photon microscope (2p-*vivo*, Intelligent Imaging Innovations, Inc., Denver) using a 20x objective. Images were acquired at 9 Hz (external pressure and paralyzed fish experiments) or 11 Hz (tail-free experiments).

Experiments on paralyzed larvae: Larvae were paralyzed by injecting 0.5 nl of 0.5 mM α-Bungarotoxin in the ventral axial musculature (Tocris, Bristol, UK) and placed in artificial cerebrospinal fluid solution (ACSF, concentrations in mM: 134 NaCl, 2.9 KCl, 1.2 MgCl_2_, 10 HEPES, 10 glucose and 2.1 CaCl_2_; 290 mOsm, adjusted to pH 7.7–7.8 with NaOH)^38^. Ventral nerve root activity was recorded with ∼10 μm tip diameter pipettes filled with ACSF. Data was recorded in current clamp mode using a MultiClamp 700 A amplifier (Molecular Devices–Axon Instruments, USA), a Digidata series 1322 A digitizer (Axon Instruments, USA) and pClamp 8.2 software (Axon instruments, USA). 10 ms water jet at 8–15 psi delivered through a pipette located in the vicinity of the otic vesicle induced reliable escapes.

Experiments on tail-free larvae: larvae were embedded dorsally and the tail was freed so that ∼1/3 remained fixed in the agar. An 890 nm LED was used to illuminate the fish from the side and behavior was recorded at 675 Hz from the bottom through a 5x microscope objective and imaged on the camera with a 50 mm camera objective. Infrared light from the imaging laser was blocked with a filter located in front of the camera. Behavior was induced as described above. Three trials per imaging position were recorded at ∼1 min inter trial intervals and the one with the clearest unilateral escape responses were selected for analysis. For each larva, one dorsal and one ventral Z-position was recorded with the agar boundary of the free tail placed roughly in the middle of the field of view to compromise between tail deflection and motion artifact. After each experiment, the larva was anesthetized with 0.02% MS-222 and a high-resolution z-stack was recorded to identify cell body position.

Passive tail bending experiments: After embedding, roughly half of the larval tail was freed unilaterally to provide access to a blunt 50 μm diameter glass probe. Probe deflections were driven with a mechanotransducer device controlled through LabView software. Tail bend was induced by probe deflection and repeated 10 times.

### Calcium imaging data analysis

Regions of interest around CSF-cN cell bodies expressing both GCaMP and tagRFP were manually defined in the software ImageJ. To extract raw fluorescence from the image time series in moving samples (either due to passive mechanical stimulation or active tail movement), the ROIs were shifted with the moving cell using a custom-made tracking algorithm based on cross correlation. In frames where the normalized correlation coefficient fell underneath a given threshold due to the movement artifact, data were omitted for that given frame. This threshold was manually adjusted to minimize tracking errors for each imaging time series. Any Δ*F*/*F* values were calculated as Δ*F*/*F*=(*F*(*t*)−*F*_0_)/(*F*_0_−*F*_*bg*_) with *F*_0_ being the average fluorescence of the 10 first frames and *F*_*bg*_ being the average fluorescence of the 10 first frames in a region with only background signal. To correct for motion artifacts due to cells moving out of the focal plane or incomplete tracking, we calculated the ratio of the GCaMP and the tagRFP signal (Δ*R*/*R*) as *ΔR*/*R*=(*F*_GCaMP_(*t*) × *F*0_*tagRFP*_/*F*0_*GCaMP*_ × *F*_*tagRFP*_(*t*))−1 with *F*0 being the average fluorescence of the 10 first frames and both *F*0 and *F*(*t*) background corrected signals (see Fig. 3a′ and extended data Figure S2). Difference in Δ*R*/*R* due to stimulation was then calculated as *difference*=(Δ*R*/*R*)_post_−(Δ*R*/*R*)_pre_ with (Δ*R*/*R*)_pre_ and (Δ*R*/*R*)_post_ being the average five time points before and after stimulation (external pressure experiments) or the average three time points before and after tail bending (tail free experiments). During motion of the tail, cells can usually not be imaged so between (Δ*R*/*R*)_pre_ and (Δ*R*/*R*)_post_ there is a gap of 4 frames (external pressure experiment) or 1 frame (tail free experiment).

### Behavior setup and analysis

To assess fish behavior, a video recording chamber was constructed. 6 dpf larvae from *Tg(pkd2l1*^*icm02/+*^*,-pkd2l1:GCaMP5G)* or *Tg(pkd2l1:Gal4, UAS:BoTxBLC-GFP)* incrosses were placed in circular swim arenas (Bioptech) atop a plexiglass plate. An Arduino Due software circuit (Arduino) was used to trigger one second-long recordings on a high speed camera (Vieworks) operating at 650 fps. To trigger escape behaviours, a 500 Hz, 10 ms sine wave stimulus was played through a 20 W amplifier (Adafruit) over two speakers (Monacor) at maximum volume 200 ms into the recording. Larvae were allowed to acclimate to the chamber for 5 min prior to the experiment and were allowed to rest 2 min between each stimulus. Each larva was subjected to five trials, the properties of which were averaged within subjects. Larvae were genotyped after data acquisition. Sample sizes were selected by performing a Lehr analysis on preliminary data sets with a threshold of 5% difference in means.

Two software packages were used to extract kinematic parameters from high-speed videos of fish during escapes. The first executable, developed in collaboration with R&D Vision, fits a series of eleven points to the fish body—two for each of the eyes, one for the swim bladder, and the remaining eight to the tail. The program identifies these 11 points for each of the 650 frames of video. The second executable consists of a series of MATLAB scripts that derives kinetic parameters from these points. First, the tail angle *α* was calculated by identifying the midpoint of the two eye points, then calculating a head direction vector based on this point and the center of the swim bladder. A tail direction vector based on the last point on the tail minus the swim bladder was then calculated, and *α* was calculated as the inverse cosine of these two vector lengths. To remove noise due to tracking error, the raw trace of *α* was median-filtered, linearly interpolated, and finally smoothed using a smoothing spline method in MATLAB's Curve Fitting Toolbox. Simultaneously, the unprocessed tail angle trace was differentiated to estimate angular velocity, which was smoothed as above. The angular velocity trace was used to define frames of movement—any frame with angular velocity >1°/frame is defined as ‘in motion'. These frames were then connected into movement bouts using morphological closing with a block size of 10 frames. Detected bouts were then assigned priority weights by their distance from stimulus onset to identify the escape. Non-escape bouts were discarded and not analyzed. Next, local maxima and minima corresponding to bend amplitudes were identified within the escape bout by calculation of regional differences (see http://www.billauer.co.il/peakdet.html). These peaks were then marked as sequential (if followed by a peak in the opposite direction, that is, a bend to the other side) or non-sequential. A hemicycle for each peak was calculated as the number of frames from each peak to the next in sequence, regardless of direction. Each peak therefore receives a maximum angle, hemicycle duration, and is flagged as being either positive (in the direction of the C-bend) or negative (in the direction of the counter bend), and either sequential or non-sequential. To improve accuracy, detection of the bout onset was modified from the initial approach, which used a single threshold of the angular velocity. A straight line segment was fit to the ascending phase of the first bend, and a single-term exponential fit was calculated between this segment and a straight horizontal segment at *y*=0 between the stimulus onset and the intercept of the rising phase segment with the *y*-axis. Escape onset was then defined as the frame at which the resulting exponential fit is greater than a 2° threshold. This method reliably identified a movement onset that was independent of the amplitude of the first bend as well as resistant to noise in the angle trace. From these data, escapes were defined as those movement bouts occurring within 30 ms of the stimulus and in which the first bend is >60° (that is, a C-bend). Movement bouts failing these inclusion criteria are not analyzed. From the inputs described above we are able to derive latency (escape onset-time of stimulus presentation), bout duration (escape offset-escape onset), bout distance (travel distance of point corresponding to the swim bladder), bout speed (distance/duration), number of oscillations (sum of detected positive and negative peaks/2), C-bend amplitude (amplitude of first detected peak). Mean tail beat frequency was calculated as the inverse of two times the mean hemicycle of sequential peaks. MATLAB package is available on request; the tail-tracking algorithm is property of R&D Vision.

### Zebrafish dissociated cell cultures

Dissociated cells from 4 dpf *Tg(pkd2l1:GCaMP5G)* and *Tg(pkd2l1:tagRFP)* larvae were plated acutely in zebrafish ACSF (see Ca^2+^ imaging section) on plastic petri dishes based on a published protocol^39^. Dissociated CSF-cNs were identified based on their GCaMP5G/tagRFP fluorescence and imaged three hours after platting.

### Statistical analysis

All values are mean±s.e.m. unless otherwise noted. To account for the fact that all our data are intrinsically nested (multiple measurements of cells and/or repetition of trials within the same fish), we used linear mixed models for all our hypothesis testing^40^. All models were calculated using the freeware R using the ‘nlme' package. Detailed analysis scripts are available upon request.

## Additional information

**How to cite this article:** Böhm, U. L. *et al.* CSF-contacting neurons regulate locomotion by relaying mechanical stimuli to spinal circuits. *Nat. Commun.* 7:10866 doi: 10.1038/ncomms10866 (2016).

## Supplementary Material

Supplementary Information Supplementary Figures 1-5, Supplementary Table 1 and Supplementary References

Supplementary Movie 1 A CSF-cN in vivo showing a beating cilium in a 4 dpf larvae.

Supplementary Movie 2 A CSF-cN in vitro on a cover slip showing a beating cilium three hours after plating.

Supplementary Movie 3 Calcium imaging, cell position and simultaneous recording of locomotor behavior in Tg(pkd2l1:GCaMP5, pkd2l1:tagRFP).
