# Supplementary material for: CSF-contacting neurons regulate locomotion by relaying mechanical stimuli to spinal circuits
Source: Nat Commun. 2016 Mar 7;7:10866. doi: 10.1038/ncomms10866 (PMC4786674; doi:10.1038/ncomms10866)
Supplement: Supplementary Information — Supplementary Figures 1-5, Supplementary Table 1 and Supplementary References [file ncomms10866-s1.pdf]

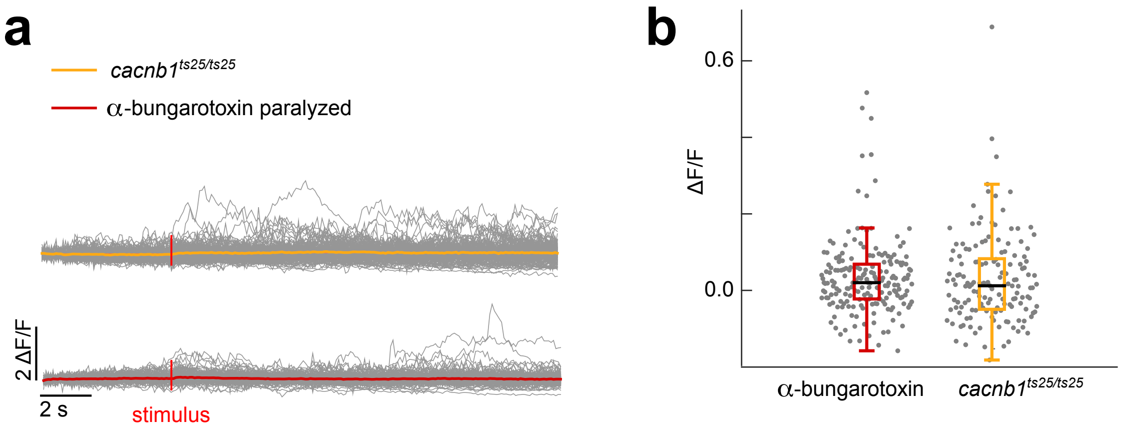

Supplemental Figure 1

CSF-cNs recorded from larva paralyzed with α-bungarotoxin and *cacnb1* mutant larva show no difference in activity. **a,** Calcium transients recorded in CSF-cNs from larva paralyzed with α-bungarotoxin (red) and in CSF-cNs from *cacnb1*$^{ts25/ts25}$ mutant larva (orange) during fictive escapes; "stimulus" indicates when the water jet was triggered, average response in colored lines, the α-bungarotoxin dataset is the same as shown in Figure 2. **b,** Quantification of calcium transient ΔF/F amplitude (each data point represents one recording from one cell; plots use median as the measure of central tendency). Responses show no significant difference between both populations (p = 0.48, 149 CSF-cNs from 12 *cacnb1*$^{ts25/ts25}$ mutant larvae: mean ΔF/F = 0.029; 192 CSF-cNs from 7 α-bungarotoxin paralyzed larvae: mean ΔF/F = 0.042).

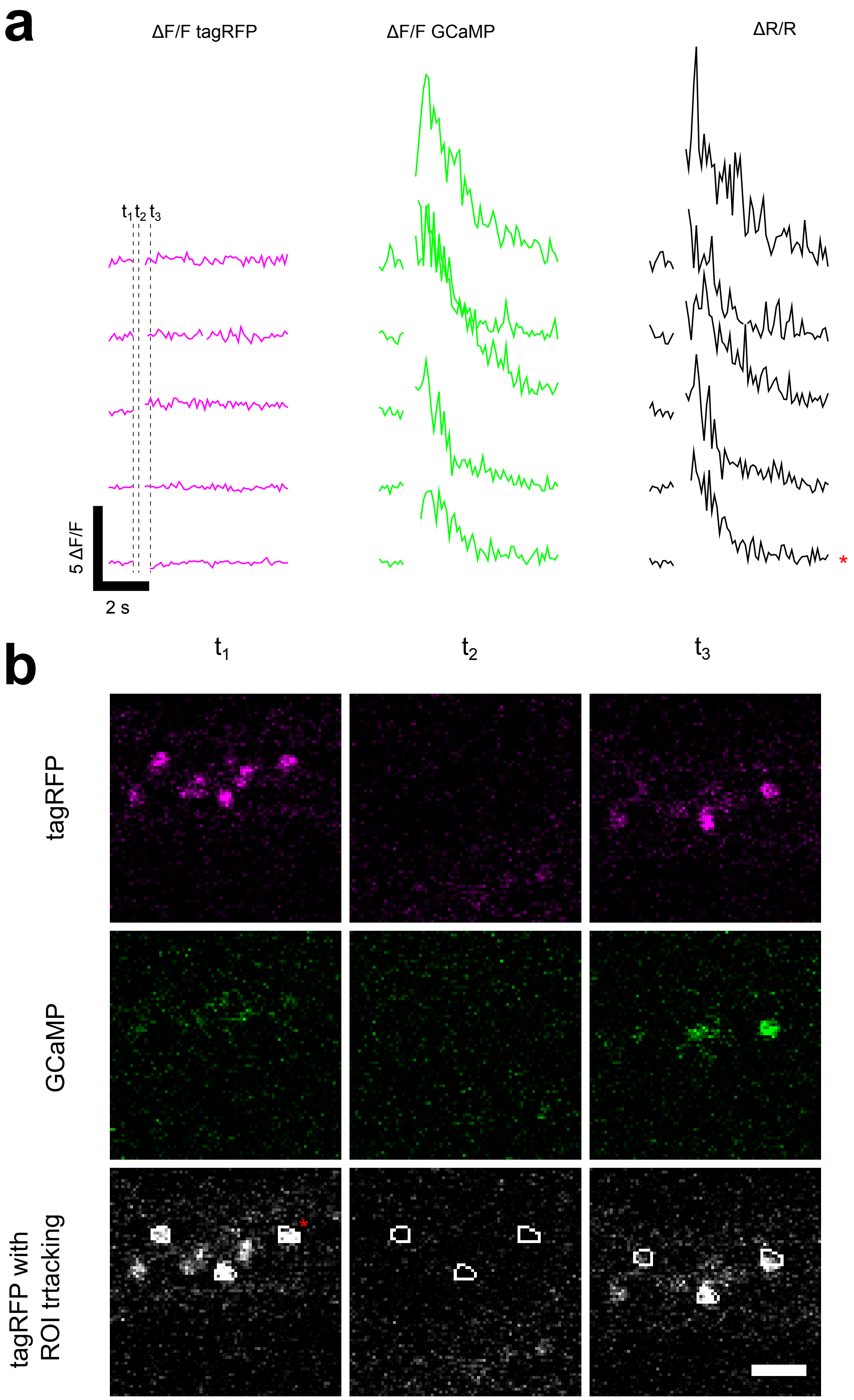
a
ΔF/F tagRFP
ΔF/F GCaMP
ΔR/R
t1 t2 t3
5 ΔF/F
2 s
*
b
t1
t2
t3
tagRFP
GCaMP
tagRFP with
ROI trtacking
*

Supplemental Figure S2

Motion artifact removal for imaging calcium transients from CSF-cNs associated with active and passive tail bends. **a,** Sample traces of responsive dorsal ipsilateral CSF-cNs from the external pressure dataset. Shown are the ΔF/F traces of the red channel (tagRFP) and the green channel (GCaMP) as well as the ΔR/R of the combination of both channels to accounting for the motion artifact. The gap represents the time points immediately after the onset of stimulation where large movements preclude imaging of the cell. The ΔF/F is used only for illustration purposes and the ΔR/R is calculated from the raw, background corrected signal. **b,** Sample images of the three time points indicated in **a**. At t_1, GCaMP fluorescence is very low while increased fluorescence can be seen at t_3 for the cell body that returns to the focal plane. The change in fluorescence due to shifts in focal plane is estimated from the tagRFP signal. The tagRFP signal is also used to track the position of the cell body in order to move the corresponding region of interest (ROI). During the stimulation (t_2) cells move out the focal plane and tracking fails. These data points are omitted in the analysis. The asterisk marks the ROI, which corresponds to the sample trace in **a**. The sample data shown is from passive external pressure experiments but the principle of analysis is the same for the tail free experiments. Scale bar: 20 µm.

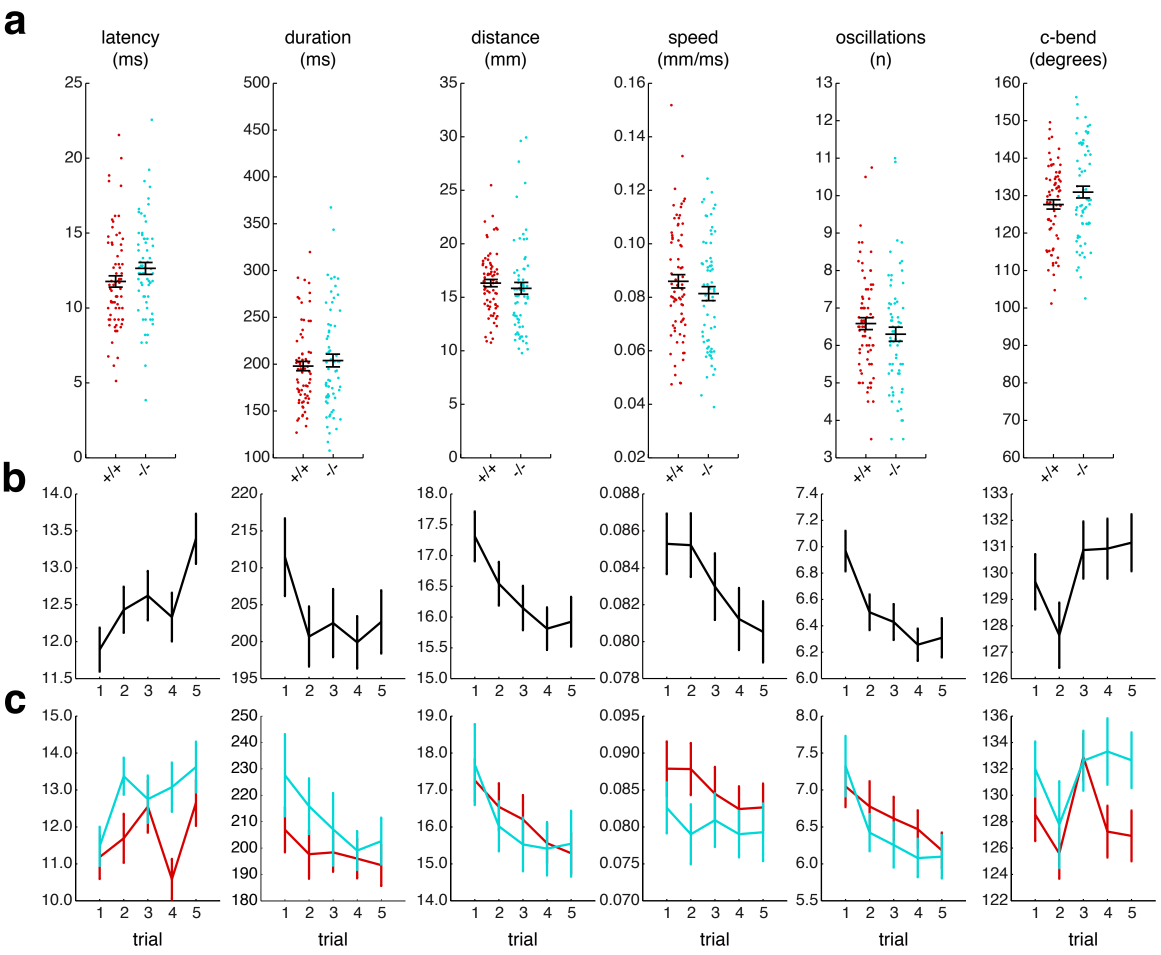

Supplemental Figure 3

Additional behavioral parameters are not affected in *pkd2l1*$^{icm02/icm02}$ mutant larvae. **a,** Latency, duration, distance, speed, number of oscillations and C bend amplitude were not affected in the

mutant. **b,** Two minute inter trial intervals led to an increase in latency (p = 4.1x10^{-3}) and a reduction of distance (p = 4.1x10^{-3}), speed (p = 0.015), and number of oscillations (p = 8.1x10^{-4}). Escape duration and C bend amplitude were the only parameters not affected by this apparent habituation in our assay. **c,** Same as in **b** with wildtypes (240 escapes from 73 larvae) in red and *pkd2l1* mutants (217 escapes from 66 larvae) in blue.

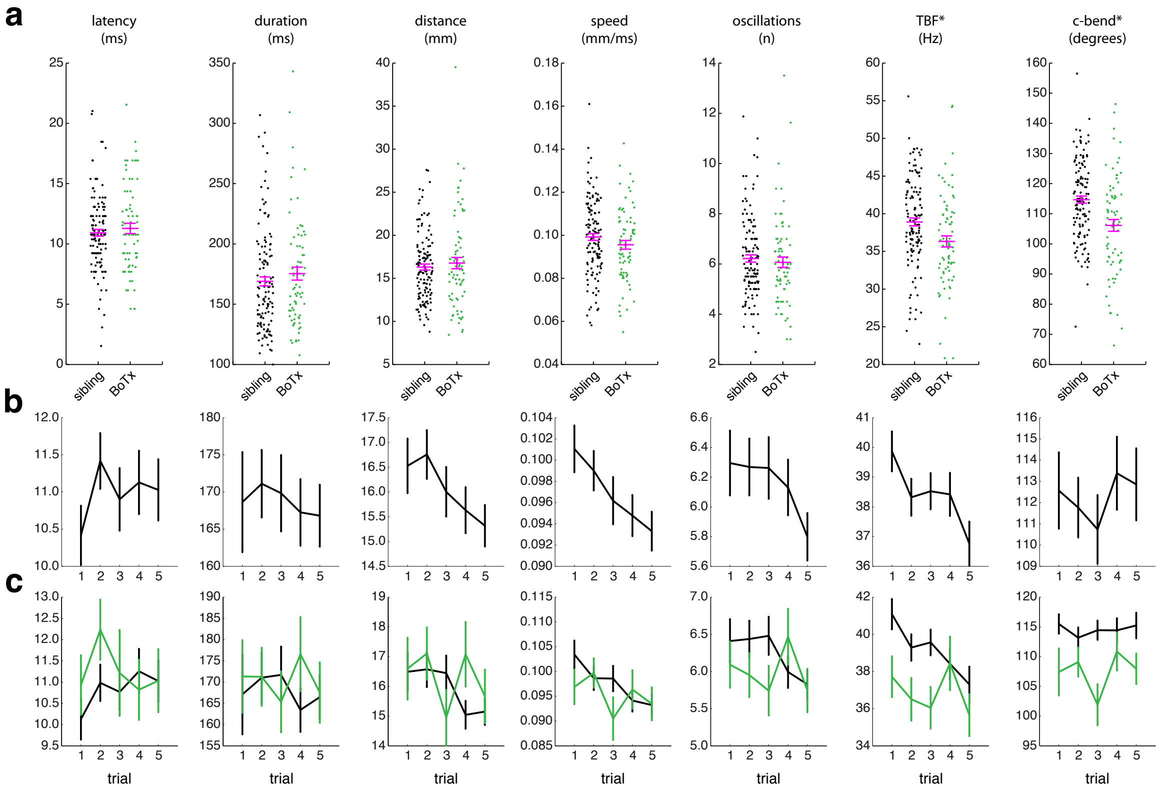

Supplemental Figure 4

Silencing of vesicular release in CSF-cNs by Botulinum toxin (BoTx) causes behavioral deficits reminiscent of $pkd2l1^{icm02/icm02}$ larvae, in particular, a reduction of TBF. **a,** TBF is significantly reduced in BoTxLC-GFP $^{+}$ larvae (p = 0.0042), as is C bend amplitude (sibling mean: 114.68 ± 1.17°, BoTxLC-GFP $^{+}$ mean: 106.13 ± 1.95°, p = 0.0001). **b,** Two minute inter trial intervals lead to a significant decrease in speed (p = 0.01) and TBF (p = 0.01). Other parameters exhibit trends suggesting habituation, but none are statistically significant. **c,** Same as in **b**, siblings represented in black (368 escapes from 128 larvae) and BoTxLC-GFP $^{+}$ larvae in green (177 escapes from 74 larvae). Accounting for the habituation effects observed in **b**, significance increases for the TBF effect (p = 0.0025).

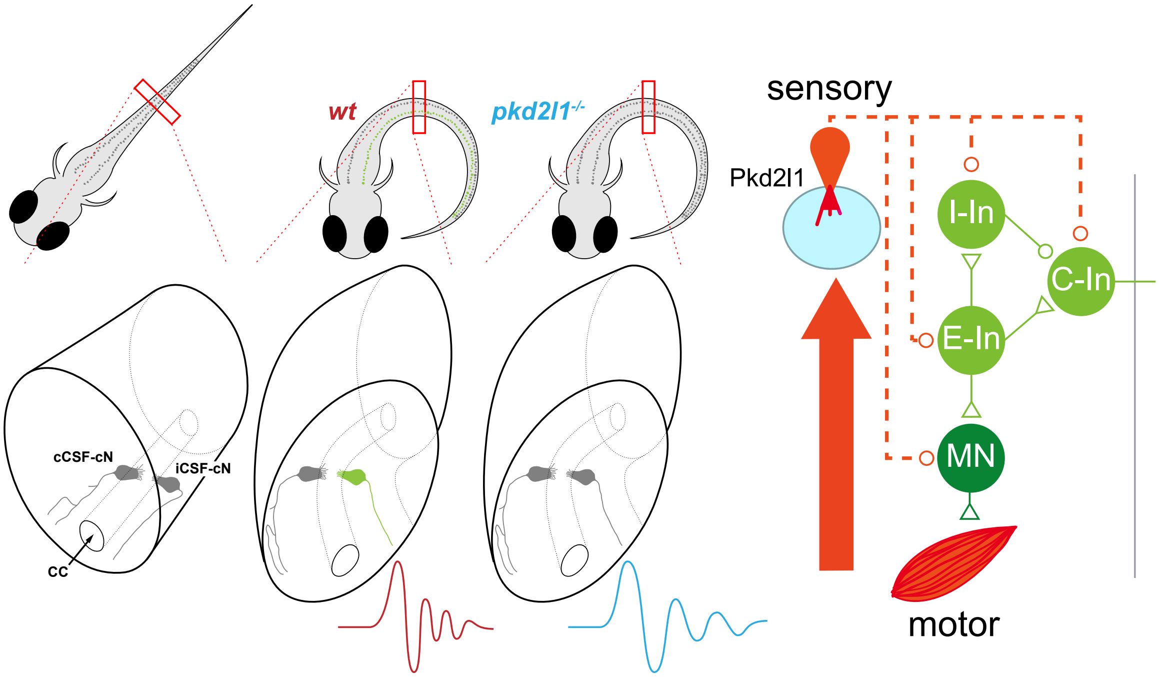

Supplemental Figure 5

Cerebrospinal fluid-contacting neurons (CSF-cNs) are located on either side of the central canal in the spinal cord. These neurons are recruited during innate locomotion when the spinal cord is bending, specifically on the compressed side. During movement, CSF-cNs provide a GABAergic mechanosensory feedback input to the locomotor central pattern generators that modulates locomotor frequency.

Supplemental Table 1

Transgenic lines and mutants generated for and used in this study.

Transgenic/mutant	Affected gene	Description	Original reference
Tg(pkd2l1:GCaMP5G)icm07		A promoter fragment of the *pkd2l1* gene drives expression of the calcium indicator GCaMP5G in CSF-cNs.	Generated in this study
Tg(UAS:GCaMP6f;cryaa:mCherry)icm06		The expression of the calcium indicator GCaMP6f is dependent of the activation of a 5' upstream activator sequence (UAS). A crystalline promoter fragment (*cryaa*) induces expression of mCherry in the lens.	Generated in this study
Tg(pkd2l1:Gal4)icm10		A promoter fragment of the *pkd2l1* gene drives expression of a GFF (optimized Gal4) transactivator in CSF-cNs	[1]
$pkd2l1^{icm02}$	*pkd2l1*	The mutant $pkd2l1^{icm02}$ was generated with TALE nucleases engineered against the second exon of *pkd2l1*. +1 frameshift.	Generated in this study
Tg(mnx1:Gal4)icm23		The *Tg(mnx1:gal4)* line driving selective expression in spinal motor neurons based on the injection of the *mnx1* construct [2].	Generated in this study
Tg(UAS:tagRFP-caax;cmcl2:eGFP)icm22		The expression of tagRFP localized to the membrane with a CAAX motif is dependent of the activation of a 5' upstream activator sequence (UAS). A crystalline promoter fragment (*cryaa*) induces expression of eGFP in the lens.	Generated in this study
Tg(pkd2l1:tagRFP)icm17		A promoter fragment of the *pkd2l1* gene drives expression of tagRFP in CSF-cNs.	Generated in this study
Tg(βact:Arl13-GFP)		A promoter fragment of the *βact* gene drives expression of the mouse Arl13b open reading frame fused to GFP.	[3]
$relaxed^{ts25}$	*cacnb1*	A point mutant of *cacnb1* generated by ENU mutagenesis causing a premature stop codon in exon 13.	[4]
Tg(UAS:BoTxBLC-GFP)icm21		Botulinum toxin light chain serotype B codon-optimized for zebrafish with C-terminal GFP fusion.	Generated in this study

Supplemental References

1. Fidelin, K. *et al.* State-Dependent Modulation of Locomotion by GABAergic Spinal Sensory Neurons. *Curr Biol* **25,** 3035–3047 (2015).
2. Zelenchuk, T. A. & Brusés, J. L. In Vivo labeling of zebrafish motor neurons using an mnx1 enhancer and Gal4/UAS. *genesis* **49,** 546–554 (2011).
3. Borovina, A., Superina, S., Voskas, D. & Ciruna, B. Vangl2 directs the posterior tilting and asymmetric localization of motile primary cilia. *Nat Cell Biol* **12,** 407–412 (2010).
4. Granato, M. *et al.* Genes controlling and mediating locomotion behavior of the zebrafish embryo and larva. *Development* **123,** 399–413 (1996).
